# Subdiaphragmatic bronchogenic cysts: Case series and literature review

**DOI:** 10.3389/fmed.2022.993091

**Published:** 2022-10-05

**Authors:** Jianchun Xiao, Xueyang Zhang, Hongru Zhou, Tao Hong, Binglu Li, Xiaodong He, Wei Liu

**Affiliations:** ^1^Department of General Surgery, Peking Union Medical College Hospital, Beijing, China; ^2^Tsinghua University School of Medicine, Beijing, China

**Keywords:** bronchogenic cyst, subdiaphragmatic, subdiaphragmatic bronchogenic cyst, case series, literature review

## Abstract

Bronchogenic cysts are congenital malformations caused by aberrant foregut budding. They major occur in the thorax, with subdiaphragmatic cases being uncommon. Here, we present a series of 19 patients diagnosed with subdiaphragmatic bronchogenic cysts histopathologically at a single institution in China from 2012 to 2021. A literature review was also conducted by searching the PubMed database using keywords related to “bronchogenic cysts” and “subdiaphragmatic,” yielding 107 cases. Taken together, the 126 cases had a median age of 41.0 years (interquartile range, 30.0–51.0 years) and 62 of them were male (49.2%). The cysts were most commonly detected in the left adrenal region (36.2%), followed by the pancreatic region (11.5%) and gastric cardia/lesser curvature of the stomach (9.2%). All patients except two underwent surgery for a definite diagnosis, symptom alleviation, and (or) malignancy prevention. Most patients recovered fast and were discharged from the hospital within 1 week after surgery, and the surgical complications were infrequent. The prognosis was generally favorable, as no recurrence was reported during the follow-up as long as 77 months.

## Introduction

Bronchogenic cysts are congenital cysts caused by aberrant budding of the primitive foregut or tracheobronchial tree. They usually locate in the thorax, especially in the mediastinum (1–3). However, they can be found in various atypical locations along the developmental pathway of the foregut, ranging from the pharynx to the spinal canal (4, 5). Bronchogenic cysts can occur infrequently below the diaphragm, with the majority located in the retroperitoneal space, particularly the left adrenal region. Subdiaphragmatic bronchogenic cysts (sBCs) can be asymptomatic, and thus are usually recognized occasionally by imaging analyses. Nevertheless, symptoms, such as fever, abdominal pain, and nausea, appear when the cysts become infected or expand sufficiently to compress nearby organs (6, 7). Due to their rarity and non-specific imaging presentation, sBCs are frequently misdiagnosed, and only histopathological examination can currently provide a definitive diagnosis. Additionally, though generally benign, malignant transformation was observed in several cases (8). Therefore, up to now, surgical resection has been the only therapeutic strategy to alleviate symptoms, establish a definitive diagnosis, and prevent malignant transformation in patients with sBCs.

Despite the fact that cases of sBCs have been previously reported (6, 7), the sample size was generally small, leading to an obscure understanding of the epidemiology, clinical presentation, diagnosis, and management strategy, et al. Here, we aim to provide a better comprehension of sBCs by presenting a series of 19 patients from a single institution as well as a review of related articles.

## Subjects and methods

All cases of sBCs from 2012 to 2021 at Peking Union Medical College Hospital in China were retrospectively identified from the hospital information system. Only patients with histopathological diagnoses of sBCs were included in the analysis. Parameters, such as demographic characteristics, clinical presentation, imaging findings, surgical information, and histopathological examination, were extracted from electronic medical records. All patients were contacted by phone in May 2022 for information on recurrence and surgical complications.

For the literature review, PubMed was searched in May 2022 using the following keywords: “bronchogenic cyst*”, “bronchial cyst*,” “subdiaphragm*,” “retroperitoneal,” and “abdom*.” The detailed search strategy can be found in Supplementary Appendix 1. The inclusion criteria of studies were full-text English articles reporting patient(s) diagnosed as sBCs histopathologically with detailed demographic and clinical information. Unpaired *t*-test or Pearson’s chi-square test was employed when appropriate. All statistical analyses were performed using SPSS 22.0.

## Case series

**FIGURE 1:**
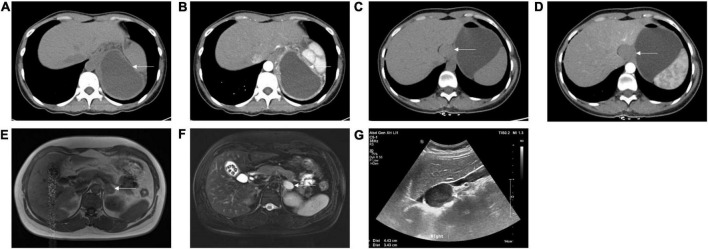
Appearance of subdiaphragmatic bronchogenic cysts on computed tomography (CT), magnetic resonance imaging (MRI), and B ultrasound. **(A)** Non-enhanced CT showed a low-density cystic lesion between the pancreatic tail, stomach, spleen, and diaphragm from case 13. **(B)** Enhanced CT showed slight enhancement of the cystic wall from case 13. **(C)** Non-enhanced CT showed a soft-tissue density lesion adjacent to the lesser curvature of the stomach from case 19. **(D)** Enhanced CT showed no enhancement of the lesion from case 19. **(E)** T1 weighted image on MRI showed a hypointense lesion at the left adrenal region from case 16. **(F)** T2 weighted image on MRI showed a hyperintense lesion at the left adrenal region from case 16. **(G)** B ultrasound showed a well-defined, hypoechoic lesion at the right adrenal region from case 17. Arrows indicate the cysts.

Nineteen patients (4 [21.1%] male; median age, 44.0 years [interquartile range, 35.0–46.5 years]) with histopathological diagnoses of subdiaphragmatic bronchogenic cysts from 2012 until 2021 were identified. Patient demographics are depicted in Table 1. Fourteen patients (73.7%) were asymptomatic and discovered the cyst by accident, either during a regular check-up (*n* = 12) or a radiographic examination done for unrelated indications (*n* = 2). Among the symptomatic patients (*n* = 5), two complained of flank pain, one of abdominal pain, one of coughing, and the other of acid reflux and abdominal distention. In all patients, the cyst was solitary with a median diameter of 5.1 cm (interquartile range [IQR]: 3.6-6.6 cm). The cysts were inclined to locate in the left abdomen (15/19, 78.9%) rather than the midline or right abdomen, the upper abdomen (16/19, 84.2%) instead of the lower abdomen, and the retroperitoneal space (16/19, 84.2%) compared with intra-abdomen. The left adrenal region (7/19, 36.8%) was the most common location, followed by the pancreatic region (3/19, 15.8%) and gastric cardia/lesser curvature of the stomach (3/19, 15.8%). On computed tomography (CT), the density of cysts ranged from low to slightly high. The number of cysts with low to slightly low, soft-tissue, and slightly high density on CT is eight, nine, and two, respectively. Only three patients underwent magnetic resonance imaging (MRI), and all of their cysts were hypointense on T1 weighted image. As for T2 weighted image, two of them were hyperintense while the other was hypointense. Twelve of 19 cysts (63.2%) showed no enhancement on CT or MRI. Ten patients received the B ultrasound examination, displaying an anechoic (*n* = 3), hypoechoic (*n* = 3), or mix-echoic (anechoic to hypoechoic) cyst (*n* = 4). Three of the cysts were multilocular with septa present. The representative appearance of the cysts on CT, MRI, and ultrasound is demonstrated in Figure 1. Due to their rarity, none of the patients were diagnosed with sBCs before the surgery.

**TABLE 1:** Demographics, clinical symptoms, imaging features, and preoperative diagnosis of 19 patients with subdiaphragmatic bronchogenic cyst from our case series.

Case	Year	Age (years)	Sex	Clinical symptoms	Cyst number	Cyst localization	Size (cm)	CT features	Other imaging studies	Preoperative diagnosis
1	2012	52	Female	None	1	Left adrenal region	2.7	Slightly high density, no enhancement	NA	Adrenal adenoma
2	2012	39	Male	None	1	Left adrenal region	7.7	Soft-tissue density, slight enhancement	SRS: negative	Adrenal adenoma
3	2013	30	Female	None	1	Left adrenal region	3.7	Slightly low density, slight enhancement	BUS: well-defined, hypoechoic to anechoic	Adrenal adenoma
4	2014	44	Female	None	1	Left diaphragm	5.0	Low to iso density, no enhancement	NA	Benign lesion
5	2014	45	Female	Flank pain	1	Left diaphragm	6.4	Soft-tissue density, no enhancement	SRS: negative	Adrenal ganglioneuroma
6	2015	45	Female	Flank pain	1	Left adrenal region	3.5	Soft-tissue density.	BUS: well-defined, hypoechoic MRI: hypointense on T1WI, hypointense on T2WI, and enhancement around the boundary. SRS: negative	Adrenal adenoma
7	2015	37	Male	None	1	Pancreatic region	6.8	Soft-tissue density, no enhancement	BUS: anechoic, with septa.	Gastrointestinal stromal tumor
8	2016	62	Male	None	1	Lesser curvature of stomach	4.7	Slightly low density, no enhancement	BUS: hypoechoic to anechoic	Gastrointestinal stromal tumor
9	2016	48	Female	None	1	Left adrenal region	4.1	Soft-tissue density, no enhancement	MIBG scan: negative	Adrenal adenoma
10	2017	44	Female	None	1	Presacral space	5.1	Soft-tissue density, heterogeneous enhancement	BUS: anechoic, with septa	Colorectal carcinoma
11	2017	39	Male	None	1	Left adrenal region	3.5	Low density, no enhancement	NA	Adrenal ganglioneuroma
12	2018	62	Female	Abdominal pain	1	Lesser curvature of stomach	6.0	Soft-tissue density, no enhancement	NA	Gastrointestinal stromal tumor
13	2018	31	Female	Cough	1	Pancreatic tail	9.4	Low density, enhancement at cyst wall	BUS: mixed echo	Mucinous cystic neoplasms
14	2019	30	Female	None	1	Presacral space	7.4	Low density, slight enhancement	BUS: anechoic, with septa MRI: hypointense on T1WI, and hyperintense on T2WI	Epidermoid cyst
15	2020	44	Female	None	1	Posteroinferior to the left kidney	8.9	Low density, no enhancement	NA	Gastrointestinal stromal tumor
16	2020	36	Female	None	1	Left adrenal region	2.5	Low density, slight enhancement.	BUS: hypoechoic MRI: hypointense on T1WI, and hyperintense on T2WI	Adrenal adenoma
17	2020	34	Female	None	1	Right adrenal region	5.5	Low density, no enhancement	BUS: well-defined, hypoechoic SRS: negative	Adrenal ganglioneuroma
18	2020	65	Female	None	1	Pancreatic tail	6.0	Slightly high density, no enhancement	BUS: well-defined, hypoechoic to anechoic	Lymphocyst
19	2021	29	Female	Acid reflux and abdominal distension	1	Lesser curvature of stomach	3.4	Soft-tissue density, no enhancement	NA	Gastrointestinal stromal tumor

BUS, B ultrasound; CT, computed tomography; MIBG, meta-iodobenzylguanidine; MRI, magnetic resonance imaging; NA, not available; SRS, somatostatin receptor scintigraphy.

**FIGURE 2:**
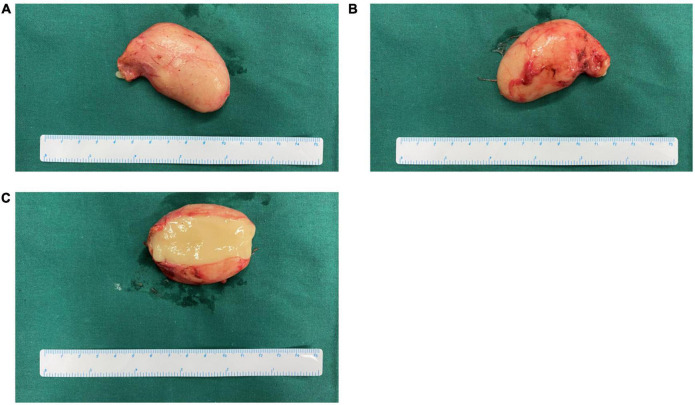
Gross appearance of the cyst from case 18 in our series. **(A,B)** Gross appearance from two sides, respectively. **(C)** Cross-section of cyst showed yellowish gelatinous content.

Laparoscopic surgery was conducted in most of the patients (16/19, 84.2%), while two underwent laparotomy and one underwent video-assisted thoracoscopic surgery (VATS; Table 2). The content of cysts was found to be either serous (1/13, 7.7%), mucoid (3/13, 23.1%), gelatinous (6/13, 46.2%), brittle (2/13, 15.4%), or mixed (1/13, 7.7%). Specifically, the gross appearance of the cyst from case 18 is depicted in Figure 2, with a cut surface demonstrating the gelatinous content within the cyst. Generally, most patients recovered fast after surgery and were discharged from the hospital within one week post-surgery. Two patients (cases 6 and 13) encountered perioperative complications and thus stayed for an extended period of time in the hospital. Case 6 had lymphatic leakage that improved with local drainage and a low-fat diet. Case 13 experienced gastroparesis and gastrointestinal decompression and acupuncture led to improvement.

**TABLE 2:** Surgical and follow-up information of 19 patients from our case series.

Case	Surgical procedure	Duration of surgery (minutes)	Content of cyst	Post-surgery hospital stay (days)	Follow-up
1	Laparoscopy	55	NA	3	NA
2	Laparoscopy	180	NA	6	NA
3	Laparoscopy	60	Gelatinous substance	4	NA
4	Video-assisted thoracoscopic surgery	110	NA	3	Last imaging examination in 2014/09 showed no signs of recurrence. Remained asymptomatic until 2022/05
5	Laparoscopy	105	Mucoid substance	6	Last imaging examination in 2021/04 showed no signs of recurrence. Underwent left nephrectomy die to left artery stenosis in 2015/09
6	Laparoscopy	55	NA	19	Perioperative lymphatic leakage. Long-term follow up not available
7	Laparotomy	105	Gelatinous substance	7	Last imaging examination in 2018/03 showed no signs of recurrence. Remained asymptomatic until 2022/05
8	Laparoscopy	95	Brittle tissue	5	Last imaging examination in 2018/06 showed no signs of recurrence. Underwent incision hernial repair in 2018/06.
9	Laparoscopy	90	Mucoid substance	5	NA
10	Laparoscopy	60	Gelatinous substance mixed with brittle tissue	7	Last imaging examination in 2022/03 showed no signs of recurrence. Remained asymptomatic until 2022/05
11	Laparoscopy	65	NA	4	Last imaging examination in 2017/07 showed no signs of recurrence. Remained asymptomatic until 2022/05
12	Laparoscopy	130	Brittle tissue	6	Last imaging examination in 2021/08 showed no signs of recurrence. Underwent incision hernial repair in 2021/08
13	Laparotomy	260	Mucoid substance	20	Perioperative gastroparesis. Long-term follow up not available
14	Laparoscopy	110	NA	7	Last imaging examination in 2020/12 showed no signs of recurrence. Remained asymptomatic until 2022/05
15	Laparoscopy	60	Serous substance	2	Last imaging examination in 2020/07 showed no signs of recurrence. Remained asymptomatic until 2022/05
16	Laparoscopy	110	Gelatinous substance	8	Last imaging examination in 2022/04 showed no signs of recurrence. Remained asymptomatic until 2022/05
17	Laparoscopy	120	Gelatinous substance	5	NA
18	Laparoscopy	95	Gelatinous substance	4	Last imaging examination in 2021/05 showed no signs of recurrence. Remained asymptomatic until 2022/05
19	Laparoscopy	85	Gelatinous substance	7	Last imaging examination in 2021/08 showed no signs of recurrence. Remained asymptomatic until 2022/05

NA, not available.

We attempted to contact all patients for information on recurrence and long-term complications in May, 2022, with 12 responses. Unfortunately, not every patient had regular imaging examinations after surgery, but they all claimed no signs of recurrence until their most recent imaging assessment, and the longest one was 77 months after surgery. As for long-term complications, two patients (cases 8 and 12) had incision hernias and both received surgery for repairment. Additionally, left renal artery stenosis and left renal atrophy were discovered 8 months after surgery in case 5 whose cyst was located in the left adrenal region. Later, this patient received a left nephrectomy.

## Literature review

194 records were retrieved initially after searching the PubMed using the strategy displayed in Supplementary Appendix 1. Among them, 49 were non-English, and the full text was not available for 21 English citations. Of the remaining 124 articles, 95 were identified to describe cases of sBCs and were included for further analysis (6, 7, 9–101). The detailed information of all included studies is displayed in Supplementary Table 1.

The 95 citations reported 107 cases (58 [54.2%] male) in total (Table 3). The median age of the cases was 41.0 years, with an IQR of 27.0–51.0 years. Specifically, the cysts could be identified as early as the prenatal stage (19, 64, 87), and were histopathologically diagnosed as sBCs later either through an autopsy after a spontaneous abortion or surgery after birth. The cases were reported from 24 countries around the world, and China is the one that described the most (25/107, 23.4%). Different from our series (5/19, 26.3%), more than half of the cases from the literature were symptomatic (59/103, 57.3%). Abdominal pain/flank pain was the most common symptom (35/103, 34.0%), and other symptoms included nausea, vomiting, and abdominal discomfort, et al.

**TABLE 3:** Literature review and our series of subdiaphragmatic bronchogenic cysts.

	Total (*n* = 126)	Literature (*n* = 107)	Our series (*n* = 19)	*p* value
**Age, years**	*n* = 126	*n* = 107	*n* = 19	0.261
Median	41.0	41.0	44.0	–
IQR	30.0–51.0	27.0–51.0	35.0–46.5	–
Range	0.0–81.0	0.0–81.0	29.0–65.0	–
**Sex**	*n* = 126	*n* = 107	*n* = 19	0.008
Male	62 (49.2%)	58 (54.2%)	4 (21.1%)	–
Female	64 (50.8%)	49 (45.8%)	15 (78.9%)	–
**Country**	*n* = 126	*n* = 107	*n* = 19	–
Asia	87 (69.0%)	68 (63.6%)	19 (100%)	–
China	44 (34.9%)	25 (23.4%)	19 (100%)	–
Europe	21 (16.7%)	21 (19.6%)	0 (0%)	–
North America	12 (9.5%)	12 (11.2%)	0 (0%)	–
Oceania	3 (2.4%)	3 (2.8%)	0 (0%)	–
South America	2 (1.6%)	2 (1.9%)	0 (0%)	–
Africa	1 (0.8%)	1 (0.9%)	0 (0%)	–
**Symptoms**	*n* = 122	*n* = 103	*n* = 19	–
Asymptomatic	58 (47.5%)	44 (42.7%)	14 (73.7%)	0.013
Symptomatic	64 (52.5%)	59 (57.3%)	5 (26.3%)	0.013
Abdominal pain/flank pain	38 (31.1%)	35 (34.0%)	3 (15.8%)	–
**Cyst number**	*n* = 126	*n* = 107	*n* = 19	0.761
1 cyst	122 (96.8%)	103 (96.3%)	19 (100%)	–
2 cysts	2 (1.6%)	2 (1.9%)	0 (0%)	–
3 cysts	1 (0.8%)	1 (0.9%)	0 (0%)	–
4 cysts	1 (0.8%)	1 (0.9%)	0 (0%)	–
**Cyst size, cm**	*n* = 130	*n* = 111	*n* = 19	0.462
Median	5.0	5.0	5.1	–
IQR	3.5–7.1	3.5–7.3	3.6–6.6	–
Range	1.5–18.9	1.5–18.9	2.5–9.4	–
**Cyst localization**	*n* = 130	*n* = 111	*n* = 19	–
Left abdomen	100 (76.9%)	85 (76.6%)	15 (78.9%)	0.892
Midline	12 (9.2%)	10 (9.0%)	2 (10.5%)	0.892
Right abdomen	18 (13.8%)	16 (14.4%)	2 (10.5%)	0.892
Upper abdomen	121 (93.1%)	105 (94.6%)	16 (84.2%)	0.126
Lower abdomen	9 (6.9%)	6 (5.4%)	3 (15.8%)	0.126
Retroperitoneal	101 (77.7%)	85 (76.6%)	16 (84.2%)	0.564
Intra-abdominal	29 (22.3%)	26 (23.4%)	3 (15.8%)	0.564
Left adrenal region	47 (36.2%)	40 (36.0%)	7 (36.8%)	–
Pancreatic region	15 (11.5%)	12 (10.8%)	3 (15.8%)	–
Gastric cardia/lesser curvature of stomach	12 (9.2%)	9 (8.1%)	3 (15.8%)	–
**Enhancement on CT/MRI**	*n* = 69	*n* = 50	*n* = 19	0.375
No	49 (71.0%)	37 (74.0%)	12 (63.2%)	–
Yes	20 (29.0%)	13 (26.0%)	7 (36.8%)	–
**Surgical procedure**	*n* = 91	*n* = 72	*n* = 19	–
Laparoscopy	58 (63.7%)	42 (58.3%)	16 (84.2%)	–
Laparotomy	25 (27.5%)	23 (31.9%)	2 (10.5%)	–
Robotic surgery	2 (2.2%)	2 (2.8%)	0 (0%)	–
Thoracotomy	2 (2.2%)	2 (2.8%)	0 (0%)	–
VATS	2 (2.2%)	1 (1.4%)	1 (5.3%)	–
**Follow-up time for recurrence, months**	*n* = 45	*n* = 33	*n* = 12	–
Median	12	12	19	–
IQR	6–24	6–24	7–30	–
Range	1–77	2–49	1–77	–

Unpaired *t*-test or Pearson’s chi-square test was employed when appropriate. CT, computed tomography; IQR, interquartile range; MRI, magnetic resonance imaging; VATS, video-assisted thoracoscopic surgery.

sBC was solitary in 96.3% (103/107) cases. One case had two cysts in the left adrenal region (62), and one case had three cysts arising from the stomach (73). In one case, the cysts were found to be bilateral, affecting both the left and right adrenal regions (66). Moreover, multiple cysts could be identified on both sides of the diaphragm, as one patient had one cyst in the left adrenal region with three in the left lung (75). The median size of the 111 sBCs reported in the literature was 5.0 cm (IQR: 3.5–7.3 cm), and could be as large as 18.9 cm (30). It should be noticed that the sBCs might enlarge over time, as manifested in several studies (19, 25, 37, 38, 50). Similar to our series, the cysts reported in the literature were more likely to be detected in the left abdomen (85/111, 76.6%), upper abdomen (105/111, 94.6%), and retroperitoneal space (85/111, 76.6%). The three most frequent areas for sBCs were the left adrenal region (40/111, 36.0%), pancreatic region (12/111, 10.8%), and gastric cardia/lesser curvature of the stomach (9/111, 8.1%).

Generally speaking, patients with sBCs usually showed normal blood tests, though abnormalities could be seen seldomly. For instance, several patients had significantly elevated serum levels of carbohydrate antigen 19-9 (CA19-9), even reaching 4330.7 U/mL. In these cases, bronchial epithelial cells were positive for CA19-9 in the immunohistochemical study on surgical specimens and the serum levels returned to normal after surgery (27, 81, 98). Interestingly, one study illustrated that the sBC was able to uptake radioiodine in the single photon emission computed tomography (SPECT) examination (68). Whether this is universal awaits further evidence.

All patients except two underwent surgery in the literature. Laparoscopy was the most common surgical procedure for patients with sBCs (42/72, 58.3%), followed by laparotomy (23/72 31.9%). One of the two patients that did not receive surgery was diagnosed with sBC based on the pathohistological examination of the specimen gained from CT-guided biopsy (56). The patient was followed up for 3 years with a CT examination every 6 months, showing a stable lesion. The other patient was diagnosed through core biopsy guided by both fine needle aspirate (FNA) and endoscopic ultrasound (EUS), but the follow-up information was absent in the article (82). Though predominantly benign, malignant transformation has been demonstrated in two cases, one with well-differentiated papillary adenocarcinoma (17) and the other with intermediate-grade neuroendocrine tumor (89). The latter patient remained asymptomatic and had no recurrence for 14 months after surgery. In addition to this, the follow-up information was also provided for another 32 cases and the longest reached 49 months. There was no sign of recurrence in any of the cases, indicating a good prognosis of sBCs.

## Discussion

Bronchogenic cysts are primitive-foregut-derived congenital cystic abnormalities that usually occur in the thorax, particularly in the mediastinum (1–3). Infrequently, they can be found in subdiaphragmatic region, and only around 100 cases were reported in English up to now based on our database searching. Our case series reported here added another 19 cases.

As displayed in three articles, sBCs can be detected even during pregnancy (19, 64, 87), but most patients discovered the cyst(s) in their thirties and forties. As for sex distribution, our study and the literature produced different results. Females predominated in our study, while males had a slightly higher proportion in the literature review. One possible reason is that the sample size of our series is insufficient, while reporting bias of the articles is an alternative explanation. As our series and the literature illustrated, most of the cysts were solitary. Only on rare ocaasions were they reported to be multiple, either unilateral or bilateral, either on the same side or the both sides of diaphragm. The size of sBCs varied greatly, ranging from 1.5 to 18.9 cm based on available data from our cases and citations, but the diameter of the cysts was mostly between 3.0 and 7.0 cm (Supplementary Table 1). When it comes to the localization, our research demonstrated that the cysts were more likely to be found in the left abdomen, upper abdomen, and retroperitoneal space. Moreover, in addition to the previously reported left adrenal region and pancreatic region (7), our analysis revealed that gastric cardia/lesser curvature of the stomach was another predilection site for sBCs.

A large proportion of patients with sBCs were asymptomatic (73.7% in our series, and 42.7% in the literature review). Symptoms caused by sBCs were usually non-specific, mainly due to complications such as local compression and infection. For example, one patient with sBC in the left adrenal region had a 5-year history of hypertension (63). After the cyst was resected completely, his blood pressure returned to normal. This patient’s hypertension might be caused by a compression on the adrenal gland, kidney, or renal artery. In one of our cases (case 13), the cystic fluid was found to be turbid and smelly during the surgery. The culture of cystic fluid was positive for *Streptococcus anginosus*, providing direct evidence of intra-cyst infection. Patients with sBCs lacked specific symptoms, which was the same for laboratory findings. The tumor markers test did not aid in the diagnosis of sBCs, as nearly all patients tested negative for them. Seldomly, the epithelial cells lining the cyst were able to produce tumor markers like CA19-9, leading to a significantly elevated serum level (27, 81, 98). Additionally, it was also difficult to differentiate sBCs from other more common subdiaphragmatic cystic lesions based on imaging examinations. The density of sBCs on CT ranged from low to high, with either enhancement or no enhancement. It could be multilocular with septa or unilocular. In summary, sBCs could not be diagnosed preoperatively as they lack a distinct clinical presentation as well as laboratory and imaging findings. Furthermore, their rarity complicates the diagnosis.

Up to now, a histopathological examination is required for the diagnosis of sBCs. Biopsy guided by CT or EUS could provide the specimen before surgery. Several cases from the literature were diagnosed based on biopsy, and two of them did not receive surgery because the patients were asymptomatic (56, 82). In particular, the lesion of one patient remained stable during the 3-year follow-up (56). According to Berger-Richardson et al., core needle biopsy of retroperitoneal lesion had low rates of both early complications and needle tract seeding (102). Given that nearly 80% of sBCs were found in retroperitoneal space and they were generally benign, it might be feasible to perform a biopsy for a diagnosis and thus avoid surgery for some of the patients with retroperitoneal bronchogenic cysts. However, it should be noticed that the biopsy might be non-diagnostic due to the sampling issue (46). Furthermore, quite a few patients had symptoms that could only be alleviated by surgery. Additionally, though rare, malignant changes were detected in two cases and only affected a portion of the cyst in both cases (17, 89). Other clinical parameters, such as symptoms, cyst size, tumor marker levels, and imaging findings, did not help predict malignancy. Therefore, it was very likely that malignancy was missed due to the sampling issue of biopsy. According to our cases and the literature, patients usually recovered quickly after surgery, with a low rate of post-surgery complications. Because of the aforementioned factors, though biopsy might help with the diagnosis of sBCs, it could not yet replace surgery at present.

After the complete cyst(s) resection, patients with sBCs generally had a good prognosis. Forty-five (*n* = 12 in our series, and *n* = 33 in the literature) patients had no recurrence during the follow-up, and the longest one reached 77 months in our series. Particularly, one patient with malignant change reported in the literature was also free of recurrence for 14 months after surgery (89).

In conclusion, our series added another 19 cases to the list of rarely reported sBCs. Together with the 107 published cases, we demonstrated that the sBCs are predisposed to locate in the left adrenal region, pancreatic region, and gastric cardia/lesser curvature of the stomach. Patients with sBCs lack specific clinical symptoms, laboratory examinations, and imaging features. Surgery is still the best management strategy for patients for a definite diagnosis, symptom alleviation, and complication prevention. The prognosis is generally favorable after complete resection of the lesion.

## Data availability statement

The raw data supporting the conclusions of this article will be made available by the authors, without undue reservation.

## Ethics statement

Ethical review and approval was not required for the study on human participants in accordance with the local legislation and institutional requirements. The ethics committee waived the requirement of written informed consent for participation.

## Author contributions

WL conceived the study. JX and XZ contributed to the case collection, discussion, literature review, and first manuscript draft. WL, HZ, TH, BL, and XH provided critical revisions. All authors contributed to the article and approved the submitted version.
