# Supplementary material for: Subdiaphragmatic bronchogenic cysts: Case series and literature review
Source: Front Med (Lausanne). 2022 Oct 5;9:993091. doi: 10.3389/fmed.2022.993091 (PMC9581013; doi:10.3389/fmed.2022.993091)
Supplement: Supplementary file 1 [file Data_Sheet_1.DOCX]

**Table S1** Literature review of subdiaphragmatic bronchogenic cysts.

| **Article #** | **Country** | **Case #** | **Age (years)** | **Sex** | **Symptoms** | **Localization** | **Size (cm)** | **Surgery** | **Follow up (months)** | **Highlights** |
| --- | --- | --- | --- | --- | --- | --- | --- | --- | --- | --- |
| 1^1^ | Japan | 1 | 59 | Male | Abdominal pain, nausea, and vomiting | Pancreatic body | 7 | Laparotomy | NA | NA |
| 2^2^ | USA | 2 | 64 | Female | Nausea, vomitting | Posterior to gastric fundus | 15 | Laparotomy | NA | Fistulous communication between cyst and gastric fundus. |
| 3^3^ | USA | 3 | 4 | Female | None | Left adrenal region | 3 | NA | NA | NA |
| 4^4^ | Germany | 4 | 12 | Male | None | Right adrenal region | 1.5 | NA | NA | NA |
| 5^5^ | Czech | 5 | 21 | Male | None | Left adrenal region | 4 | Laparotomy | 4 | NA |
| 6^6^ | USA | 6 | 44 | Male | Abdominal pain | Left adrenal region | 10 | NA | NA | NA |
| 7^7^ | USA | 7 | 46 | Female | Abdominal pain and weight loss | Left adrenal region | 4 | NA | NA | NA |
| 8^8^ | Japan | 8 | 46 | Female | Left flank pain | Left adrenal region | 8 | NA | NA | NA |
| 9^9^ | Japan | 9 | 55 | Female | Lower abdominal discomfort | Ascending colon | 10 | NA | NA | Malignant transformation. |
| 10^10^ | Korea | 10 | 30 | Male | None | Left adrenal region | 7 | NA | NA | NA |
| 11^11^ | Italy | 11 | 3 months | Male | None | Right crus of diaphragm | 4.5 | NA | 3 | Prenatal diagnosis. |
| 12^12^ | Germany | 12 | 46 | Male | None | Right adrenal region | 3.8 | NA | NA | NA |
| 13^13^ | Japan | 13 | 27 | Male | None | Left adrenal region | 8 | Laparotomy | NA | NA |
| 14^14^ | UK | 14 | 51 | Male | Abdominal pain | Left adrenal region | 5 | Laparotomy | NA | NA |
| 15^15^ | Japan | 15 | 46 | Female | None | Left diaphragm | 4 | Laparotomy | NA | NA |
| 16^16^ | Spain | 16 | 51 | Male | None | Left crus of diaphragm | 8 | NA | NA | NA |
| 17^17^ | Australia | 17 | 8 | Female | Abdominal pain | Left adrenal region | 5 | NA | 24 | NA |
|  |  | 18 | 15 | Male | Thoracic pain | Left adrenal region | 5.5 | Laparoscopy | 48 | NA |
| 18^18^ | Sweden | 19 | 38 | Male | Abdominal pain and weight loss | Pancreatic body and tail | 4.5 | NA | 24 | NA |
| 19^19^ | Japan | 20 | 42 | Male | Left flank pain | Left adrenal region | 14 | NA | NA | Increased serum level of CA19-9 and decreased to normal after surgery. |
| 20^20^ | Japan | 21 | 36 | Male | None | Left adrenal region | 5 | Laparoscopy | NA | NA |
| 21^21^ | Korea | 22 | 48 | Female | Abdominal pain and dyspepsia | Inferomedial to gallbladder | 3 | Laparotomy | 2 | NA |
| 22^22^ | Singapore | 23 | 29 | Female | None | Upper right retroperitoneal | 18.9 | Laparotomy | 12 | NA |
| 23^23^ | Korea | 24 | 62 | Female | None | Lesser curvature of stomach | 1.7 | NA | NA | NA |
| 24^24^ | Korea | 25 | 59 | Male | None | Pancreatic neck | 7 | NA | NA | NA |
| 25^25^ | Korea | 26 | 33 | Male | Left chest pain | Left diaphragm | 3 | Thoracotomy | NA | Trans-diaphragm. |
| 26^26^ | USA | 27 | 46 | Female | Left flank pain | Left crus of diaphragm | 4 | NA | NA | NA |
| 27^27^ | France | 28 | 10 | Male | None | Lesser sac | 3.4 | Laparoscopy | NA | NA |
| 28^28^ | China | 29 | 54 | Female | None | Pancreatic body | 4 | NA | NA | NA |
|  |  | 30 | 69 | Male | Abdominal discomfort | Pancreatic tail | 7 | Laparotomy | NA | NA |
| 29^29^ | Korea | 31 | 17 | Male | Abdominal pain and nausea | Pancreatic tail | 3.2 | NA | NA | NA |
| 30^30^ | Japan | 32 | 39 | Male | Low-grade fever | Left adrenal region | 4.5 | Laparoscopy | NA | NA |
| 31^31^ | China | 33 | 55 | Male | None | Left adrenal region | 4 | Laparoscopy | NA | NA |
| 32^32^ | Korea | 34 | 41 | Female | None | Left adrenal region | 4.8 | Laparoscopy | NA | NA |
| 33^33^ | Turkey | 35 | 19 months | Female | Fever | Right diaphragm | 4 | Laparotomy | 24 | NA |
| 34^34^ | Germany | 36 | 43 | Female | Hiccup and abdominal discomfort | Right diaphragm | 8 | Laparoscopy | 24 | NA |
| 35^35^ | USA | 37 | 67 | Male | None | Pancreatic region | 3.9 | Laparoscopy | NA | NA |
| 36^36^ | Turkey | 38 | 36 | Male | None | Left adrenal region | 6 | Laparotomy | NA | NA |
| 37^37^ | Japan | 39 | 43 | Male | Abdominal pain | Lesser curvature of stomach | 9 | NA | NA | NA |
| 38^38^ | Spain | 40 | 67 | Male | Low back pain | Gastric cardia | 6 | Laparoscopy | NA | NA |
| 39^39^ | Portland | 41 | 32 | Female | Abdominal pain | Pancreatic neck | 2.7 | NA | NA | Intra-abdominal abscess after surgery. |
|  |  | 42 | 44 | Male | None | Left adrenal region | 3.1 | Laparoscopy | NA | NA |
| 40^40^ | Italy | 43 | 33 | Male | None | Ileal mesentery | 5 | Laparotomy | NA | NA |
| 41^41^ | Japan | 44 | 81 | Female | None | Lesser curvature of stomach | 2.6 | Laparotomy | 40 | NA |
| 42^42^ | Korea | 45 | 56 | Female | Chest pain, cough | Right diaphragm | NA | Thoracotomy | 2 | NA |
| 43^43^ | USA | 46 | 23 | Female | None | Left adrenal region | 5.2 | Laparoscopy | NA | NA |
| 44^44^ | Belgium | 47 | 48 | Male | None | Left adrenal region | 7.5 | Laparotomy | NA | NA |
| 45^45^ | Korea | 48 | 45 | Female | None | Left adrenal region | 3.6 | NA | 6 | NA |
|  |  | 49 | 45 | Female | Abdominal discomfort | Left adrenal region | 8 | NA | 49 | NA |
|  |  | 50 | 17 | Male | Abdominal pain | Pancreatic tail | 3.5 | NA | 17 | NA |
|  |  | 51 | 62 | Female | Epigastric soreness | Gastric cardia | 3.5 | NA | 14 | NA |
|  |  | 52 | 61 | Male | None | Gastric cardia | 3.5 | NA | 6 | NA |
| 46^46^ | India | 53 | 30 | Female | Abdominal pain | Upper right retroperitoneal | 10 | Laparotomy | NA | NA |
| 47^47^ | USA | 54 | 33 | Male | Abdominal pain | Lesser sac | 6 | Laparoscopy | NA | NA |
| 48^48^ | China | 55 | 50 | Female | Left flank pain | Upper left retroperitoneal | 3 | Laparoscopy | NA | NA |
|  |  | 56 | 26 | Female | None | Left adrenal region | 4.8 | NA | NA | NA |
| 49^49^ | France | 57 | 60 | Male | None | Left crus of diaphragm | NA | None | NA | No surgery. |
| 50^50^ | Argentina | 58 | 49 | Female | Abdominal pain | Pancreatic head | 12 | NA | NA | NA |
| 51^51^ | USA | 59 | 40 | Female | Ddysphagia | Left to esophageal | 5 | Laparoscopy | 6 | NA |
| 52^52^ | Japan | 60 | 71 | Male | None | Gastric cardia | 3.2 | Laparoscopy | 12 | NA |
| 53^53^ | Portugal | 61 | 36 | Female | Abdominal pain, nausea, and vomiting | Pancreatic tail | 8 | Laparoscopy | NA | NA |
| 54^54^ | Switzerland | 62 | 42 | Female | Abdominal pain | Left adrenal region | 5 | Laparoscopy | NA | High levels of CEA in cystic fluid. |
| 55^55^ | Germany | 63 | 50 | Male | Left flank pain | Left adrenal region | 2.9 | Laparoscopy | NA | Multilocular. |
|  |  |  |  |  |  | Left adrenal region | 5 |  |  |  |
| 56^56^ | Japan | 64 | 27 | Male | Hypertension | Left adrenal region | 5.4 | Laparoscopy | 12 | NA |
| 57^57^ | Czech | 65 | 6 | Female | NA | Pancreatic body and tail | 15 | Laparotomy | 36 | Prenatal diagnosis. |
| 58^58^ | China | 66 | 30 | Female | None | Left adrenal region | 2 | Laparoscopy | NA | NA |
| 59^59^ | China | 67 | 51 | Male | Vague headache, fatigue and hypertension | Right adrenal region | 1.5 | Laparoscopy | NA  NA | Bilateral. |
|  |  |  |  |  |  | Left adrenal region | 2.1 |  |  |  |
| 60^60^ | Iran | 68 | 23 | Male | None | Upper left retroperitoneal | 13 | NA | 48 | NA |
| 61^61^ | China | 69 | 52 | Male | None | Left crus of diaphragm | 2.5 | Laparoscopy | NA | Uptake radioiodine. |
| 62^62^ | USA | 70 | 40 | Female | Abdominal pain | Left to esophageal | 3 | Laparoscopy | NA | NA |
| 63^63^ | Turkey | 71 | 25 | Female | Left flank pain | Left adrenal region | 4 | Laparoscopy | NA | NA |
| 64^64^ | Korea | 72 | 57 | Male | None | Left adrenal region | 5 | Laparoscopy | NA | NA |
| 65^65^ | China | 73 | 36 | Female | Lower back pain | Upper left retroperitoneal | 17 | Laparotomy | 16 | NA |
| 66^66^ | USA | 74 | 29 | Female | Abdominal pain | Stomach | 3 | Laparotomy | NA | Multilocular. |
|  |  |  |  |  |  | Stomach | 1.8 |  |  |  |
|  |  |  |  |  |  | Stomach | 9.2 |  |  |  |
| 67^67^ | India | 75 | 34 | Female | Heaviness in right flank | Right hypochondrium | 10 | Laparoscopy | NA | NA |
| 68^68^ | China | 76 | 8 | Male | None | Left adrenal region | 3.9 | Laparoscopy | 21 | Accompanied with 3 cysts in left lung. |
| 69^69^ | China | 77 | 24 | Male | Abdominal distension | Hepatogastric ligament | 3.8 | Laparoscopy | NA | NA |
| 70^70^ | Turkey | 78 | 42 | Male | Back pain | Left diaphragm | 9 | NA | NA | NA |
| 71^71^ | France | 79 | 36 | Male | None | Retrorectal region | NA | Laparotomy | NA | Recurrence after incomplete resection. |
| 72^72^ | Tunisia | 80 | 65 | Female | Abdominal pain | Gastric cardia | 8 | NA | 12 | NA |
| 73^73^ | China | 81 | 22 | Male | Chest pain | Left diaphragm | 15 | Laparotomy | NA | NA |
| 74^74^ | China | 82 | 48 | Female | Abdominal pain | Left adrenal region | 8 | Laparoscopy | 2 | Increased serum level of CA19-9 and decreased to normal after surgery. |
| 75^75^ | USA | 83 | 52 | Male | None | Left adrenal region | 3.1 | NA | NA | NA |
| 76^76^ | China | 84 | 78 | Male | NA | Inferior to left renal vein | 7 | Robotic | 3 | NA |
|  |  | 85 | 33 | Male | NA | Left to hepatic hilum | 4.5 | Robotic | 3 | NA |
| 77^77^ | Turkey | 86 | 38 | Female | Abdominal pain | Left adrenal region | 8 | Laparoscopy | NA | NA |
| 78^78^ | China | 87 | 39 | Male | None | Distal ileum | 6 | Laparotomy | NA | NA |
| 79^79^ | China | 88 | 18 | Female | Vomiting | Gastric cardia | NA | Laparoscopy | NA | NA |
| 80^80^ | India | 89 | 20-week fetus | Female | NA | Umbilical region | 3.5 | NA | NA | Prenatal diagnosis. |
| 81^81^ | China | 90 | 33 | Male | None | Right adrenal region | 5.9 | Laparoscopy | NA | NA |
|  |  | 91 | 27 | Male | None | Left adrenal region | 4.1 | Laparoscopy | NA | NA |
| 82^82^ | India | 92 | 44 | Male | Abdominal pain | Lesser sac | 5 | Laparotomy | 14 | Malignant transformation. |
| 83^83^ | India | 93 | 46 | Male | Dyspepsia | Anterior surface of the left psoas muscle at L5-S1 level | 4 | Laparotomy | NA | NA |
| 84^84^ | China | 94 | 41 | Female | Lumbar back discomfort | Left adrenal region | 3.9 | Laparoscopy | NA | NA |
| 85^85^ | India | 95 | 30 | Male | Abdominal pain | Right crus of diaphragm | 7 | Laparoscopy | NA | NA |
| 86^86^ | New Zealand | 96 | 39 | Male | Abdominal pain | Left adrenal region | 3 | Laparoscopy | NA | NA |
| 87^87^ | Italy | 97 | 57 | Female | Abdominal pain | Left crus of diaphragm | 6 | Laparoscopy | 12 | NA |
| 88^88^ | China | 98 | 68 | Male | Upper abdominal fullness and belching | Left hypochondrium | 10 | Laparotomy | 6 | NA |
| 89^89^ | Japan | 99 | 16 | Female | Abdominal pain, back pain, and fever | Left to abdominal aorta | 3.8 | Laparoscopy | 24 | NA |
| 90^90^ | China | 100 | 17 | Female | Abdominal pain | Left adrenal region | 2.9 | Laparoscopy | 10 | NA |
| 91^91^ | China | 101 | 53 | Female | None | Left crus of diaphragm | 3.5 | Laparoscopy | 3 | NA |
| 92^92^ | Japan | 102 | 50 | Female | Abdominal pain | Left diaphragm | 5 | VATS | NA | Increased serum level of CA19-9. IHC CA19-9 positive. |
| 93^93^ | Brazil | 103 | 66 | Female | None | Left adrenal region | 6.2 | NA | NA | High levels of CA 19-9 and amylase in cystic fluid. |
| 94^94^ | China | 104 | 27 | Male | None | Right adrenal region | 3.6 | Laparoscopy | NA | NA |
|  |  | 105 | 18 | Male | None | Left adrenal region | 7.1 | Laparotomy | NA | NA |
|  |  | 106 | 6 | Male | Abdominal pain | Left adrenal region | 8 | Laparoscopy | NA | NA |
| 95^95^ | Japan | 107 | 73 | Female | None | Left adrenal region | 2.8 | NA | NA | NA |

**Abbreviations:** CA 19-9, carbohydrate antigen 19-9; CEA, carcinoembryonic antigen; IHC, immunohistochemistry; NA, not available; VATS, Video-assisted thoracoscopic surgery.

**Appendix 1** Search Strategy

#1 "bronchogenic cyst"[MeSH Terms]

#2 "bronchogenic cyst"[Title/Abstract] OR "bronchogenic cysts"[Title/Abstract] OR "bronchial cyst"[Title/Abstract] OR "bronchial cysts"[Title/Abstract]

#3 "retroperitoneal"[Title/Abstract]

#4 #1 or #2

#5 #3 and #4

**Reference**

1. Sumiyoshi K, Shimizu S, Enjoji M, Iwashita A, Kawakami K. Bronchogenic cyst in the abdomen. Virchows Arch A Pathol Anat Histopathol. 1985;408(1):93-8. doi:10.1007/bf00739965

2. Braffman B, Keller R, Gendal ES, Finkel SI. Subdiaphragmatic bronchogenic cyst with gastric communication. Gastrointest Radiol. 1988;13(4):309-11. doi:10.1007/bf01889087

3. Swanson SJ, 3rd, Skoog SJ, Garcia V, Wahl RC. Pseudoadrenal mass: unusual presentation of bronchogenic cyst. J Pediatr Surg. 1991;26(12):1401-3. doi:10.1016/0022-3468(91)91046-2

4. Fischbach R, Benz-Bohm G, Berthold F, Eidt S, Schmidt R. Infradiaphragmatic bronchogenic cyst with high CT numbers in a boy with primitive neuroectodermal tumor. Pediatr Radiol. 1994;24(7):504-5. doi:10.1007/bf02015013

5. Resl M, Navrátil P, Krajina A. Retroperitoneal bronchogenic cyst in a young adult. Respiration. 1996;63(6):387-9. doi:10.1159/000196583

6. Doggett RS, Carty SE, Clarke MR. Retroperitoneal bronchogenic cyst masquerading clinically and radiologically as a phaeochromocytoma. Virchows Arch. 1997;431(1):73-6. doi:10.1007/s004280050071

7. Buckley JA, Siegelman ES, Birnbaum BA, Rosato EF. Bronchogenic cyst appearing as a retroperitoneal mass. AJR Am J Roentgenol. 1998;171(2):527-8. doi:10.2214/ajr.171.2.9694496

8. Itoh H, Shitamura T, Kataoka H, Ide H, Akiyama Y, Hamasuna R, et al. Retroperitoneal bronchogenic cyst: report of a case and literature review. Pathol Int. 1999;49(2):152-5. doi:10.1046/j.1440-1827.1999.00837.x

9. Sullivan SM, Okada S, Kudo M, Ebihara Y. A retroperitoneal bronchogenic cyst with malignant change. Pathol Int. 1999;49(4):338-41. doi:10.1046/j.1440-1827.1999.00869.x

10. Yang SW, Linton JA, Ryu SJ, Shin DH, Park CS. Retroperitoneal multilocular bronchogenic cyst adjacent to adrenal gland. Yonsei Med J. 1999;40(5):523-6. doi:10.3349/ymj.1999.40.5.523

11. Bagolan P, Bilancioni E, Nahom A, Trucchi A, Inserra A, Neri M, et al. Prenatal diagnosis of a bronchogenic cyst in an unusual site. Ultrasound Obstet Gynecol. 2000;15(1):66-8. doi:10.1046/j.1469-0705.2000.00022.x

12. Reichelt O, Grieser T, Wunderlich H, Möller A, Schubert J. Bronchogenic cyst. A rare differential diagnosis of retroperitoneal tumors. Urol Int. 2000;64(4):216-9. doi:10.1159/000030534

13. Murakami R, Machida M, Kobayashi Y, Ogura J, Ichikawa T, Kumazaki T. Retroperitoneal bronchogenic cyst: CT and MR imaging. Abdom Imaging. 2000;25(4):444-7. doi:10.1007/s002610000019

14. Haddadin WJ, Reid R, Jindal RM. A retroperitoneal bronchogenic cyst: a rare cause of a mass in the adrenal region. J Clin Pathol. 2001;54(10):801-2. doi:10.1136/jcp.54.10.801

15. Ingu A, Watanabe A, Ichimiya Y, Saito T, Abe T. Retroperitoneal bronchogenic cyst: a case report. Chest. 2002;121(4):1357-9. doi:10.1378/chest.121.4.1357

16. Martín R, Sanz E, de Vicente E, Ortega P, Labrador E, Paumard A, et al. Differential diagnosis of asymptomatic retroperitoneal cystic lesion: a new case of retroperitoneal bronchogenic cyst. Eur Radiol. 2002;12(4):949-50. doi:10.1007/s003300101119

17. McCrystal DJ, Borzi PA. Retroperitoneoscopic resection of retroperitoneal bronchogenic cysts. Pediatr Surg Int. 2002;18(5-6):375-7. doi:10.1007/s00383-002-0829-9

18. Andersson R, Lindell G, Cwikiel W, Dawiskiba S. Retroperitoneal bronchogenic cyst as a differential diagnosis of pancreatic mucinous cystic tumor. Dig Surg. 2003;20(1):55-7. doi:10.1159/000068851

19. Hisatomi E, Miyajima K, Yasumori K, Okamura H, Nonaka M, Watanabe J, et al. Retroperitoneal bronchogenic cyst: a rare case showing the characteristic imaging feature of milk of calcium. Abdom Imaging. 2003;28(5):716-20. doi:10.1007/s00261-003-0003-4

20. Ishizuka O, Misawa K, Nakazawa M, Nishizawa O. A retroperitoneal bronchogenic cyst: laparoscopic treatment. Urol Int. 2004;72(3):269-70. doi:10.1159/000077129

21. Kim KH, Kim JI, Ahn CH, Kim JS, Ku YM, Shin OR, et al. The first case of intraperitoneal bronchogenic cyst in Korea mimicking a gallbladder tumor. J Korean Med Sci. 2004;19(3):470-3. doi:10.3346/jkms.2004.19.3.470

22. Goh BK, Chan HS, Wong WK. A rare case of "giant" right-sided retroperitoneal bronchogenic cyst. Dig Dis Sci. 2004;49(9):1491-2. doi:10.1023/b:ddas.0000042253.60289.76

23. Song SY, Noh JH, Lee SJ, Son HJ. Bronchogenic cyst of the stomach masquerading as benign stromal tumor. Pathol Int. 2005;55(2):87-91. doi:10.1111/j.1440-1827.2005.01788.x

24. Paik SS, Jang KS, Han HX, Oh YH, Lee KG, Choi D. Retroperitoneal bronchogenic cyst mimicking pancreatic pseudocyst in a patient with colorectal cancer. J Gastroenterol Hepatol. 2005;20(5):802-3. doi:10.1111/j.1440-1746.2005.03763.x

25. Jo WM, Shin JS, Lee IS. Supradiaphragmatic bronchogenic cyst extending into the retroperitoneum. Ann Thorac Surg. 2006;81(1):369-70. doi:10.1016/j.athoracsur.2004.08.033

26. Liang MK, Yee HT, Song JW, Marks JL. Subdiaphragmatic bronchogenic cysts: a comprehensive review of the literature. Am Surg. 2005;71(12):1034-41.

27. Sauvat F, Fusaro F, Jaubert F, Galifer B, Revillon Y. Paraesophageal bronchogenic cyst: first case reports in pediatric. Pediatr Surg Int. 2006;22(10):849-51. doi:10.1007/s00383-006-1738-0

28. Wang SE, Tsai YF, Su CH, Shyr YM, Lee RC, Tsai WC, et al. Retroperitoneal bronchogenic cyst mimicking pancreatic cystic lesion. J Chin Med Assoc. 2006;69(11):538-42. doi:10.1016/s1726-4901(09)70325-9

29. Kim EY, Lee WJ, Jang KT. Retroperitoneal bronchogenic cyst mimicking a pancreatic cystic tumour. Clin Radiol. 2007;62(5):491-4. doi:10.1016/j.crad.2006.10.012

30. Minei S, Igarashi T, Hirano D. A case of retroperitoneal bronchogenic cyst treated by laparoscopic surgery. Hinyokika Kiyo. 2007;53(3):171-4.

31. Chu PY, Hwang TI, Teng TH, Lee CC. A retroperitoneal bronchogenic cyst successfully treated by laparoscopic surgery. Ann Saudi Med. 2007;27(3):199-200. doi:10.5144/0256-4947.2007.199

32. Chung JM, Jung MJ, Lee W, Choi S. Retroperitoneal bronchogenic cyst presenting as adrenal tumor in adult successfully treated with retroperitoneal laparoscopic surgery. Urology. 2009;73(2):442.e13-5. doi:10.1016/j.urology.2008.02.056

33. Elemen L, Tugay M, Tugay S, Gürcan NI, Erkus B, Gurbuz Y. Bronchogenic cyst of the right hemidiaphragm mimicking a hydatid cyst of the liver: report of the first pediatric case. Pediatr Surg Int. 2008;24(8):957-9. doi:10.1007/s00383-008-2187-8

34. Zügel NP, Kox M, Lang RA, Hüttl TP. Laparoscopic resection of an intradiaphragmatic bronchogenic cyst. Jsls. 2008;12(3):318-20.

35. Obando J, Merkle E, Bean SM. A retroperitoneal bronchogenic cyst. Clin Gastroenterol Hepatol. 2009;7(8):A24-e1. doi:10.1016/j.cgh.2008.11.011

36. Onol FF, Baytekin F, Dikbas O, Ergönenç T, Tanidir Y. A retroperitoneal bronchogenic cyst mimicking adrenal tumour in an adult: is differential diagnosis truly possible? J Clin Pathol. 2009;62(2):187-9. doi:10.1136/jcp.2008.061077

37. Shibahara H, Arai T, Yokoi S, Hayakawa S. Bronchogenic cyst of the stomach involved with gastric adenocarcinoma. Clin J Gastroenterol. 2009;2(2):80-4. doi:10.1007/s12328-008-0042-z

38. Díaz Nieto R, Naranjo Torres A, Gómez Alvarez M, Ruiz Rabelo JF, Pérez Manrique MC, Ciria Bru R, et al. Intraabdominal bronchogenic cyst. J Gastrointest Surg. 2010;14(4):756-8. doi:10.1007/s11605-009-0932-5

39. El Youssef R, Fleseriu M, Sheppard BC. Adrenal and pancreatic presentation of subdiaphragmatic retroperitoneal bronchogenic cysts. Arch Surg. 2010;145(3):302-4. doi:10.1001/archsurg.2010.12

40. Petrina A, Boselli C, Cirocchi R, Covarelli P, Eugeni E, Badolato M, et al. Bronchogenic cyst of the ileal mesentery: a case report and a review of literature. J Med Case Rep. 2010;4:313. doi:10.1186/1752-1947-4-313

41. Ubukata H, Satani T, Motohashi G, Konishi S, Goto Y, Watanabe Y, et al. Intra-abdominal bronchogenic cyst with gastric attachment: report of a case. Surg Today. 2011;41(8):1095-100. doi:10.1007/s00595-010-4398-6

42. Kim JB, Park CK, Kum DY, Lee DH, Jung HR. Bronchogenic cyst of the right hemidiaphragm presenting with pleural effusion. Korean J Thorac Cardiovasc Surg. 2011;44(1):86-8. doi:10.5090/kjtcs.2011.44.1.86

43. O'Neal PB, Moore FD, Gawande A, Cho NL, King EE, Moalem J, et al. Bronchogenic cyst masquerading as an adrenal tumor: a case of mistaken identity. Endocr Pract. 2012;18(5):e102-5. doi:10.4158/ep11186.Cr

44. Govaerts K, Van Eyken P, Verswijvel G, Van der Speeten K. A bronchogenic cyst, presenting as a retroperitoneal cystic mass. Rare Tumors. 2012;4(1):e13. doi:10.4081/rt.2012.e13

45. Choi KK, Sung JY, Kim JS, Kim MJ, Park H, Choi DW, et al. Intra-abdominal bronchogenic cyst: report of five cases. Korean J Hepatobiliary Pancreat Surg. 2012;16(2):75-9. doi:10.14701/kjhbps.2012.16.2.75

46. Parray FQ, Sherwani AY, Dangroo SA, Bisati RA, Malik NS. Retroperitoneal bronchogenic cyst mimicking hydatid liver: a case report. Case Rep Surg. 2012;2012:312147. doi:10.1155/2012/312147

47. Fernández JL, Bauza G, McAneny DB. Minimally invasive management of lesser sac bronchogenic cyst. Jsls. 2011;15(4):571-4. doi:10.4293/108680811x13176785204715

48. Cai Y, Guo Z, Cai Q, Dai S, Gao W, Niu Y, et al. Bronchogenic cysts in retroperitoneal region. Abdom Imaging. 2013;38(1):211-4. doi:10.1007/s00261-012-9909-z

49. Brient C, Muller C, Cassagneau P, Taieb D, Sebag F, Henry JF. A retroperitoneal bronchogenic cyst. J Visc Surg. 2012;149(5):e361-3. doi:10.1016/j.jviscsurg.2012.05.002

50. Kluger MD, Tayar C, Belli A, Salceda JA, van Nhieu JT, Luciani A, et al. A foregut cystic neoplasm with diagnostic and therapeutic similarities to mucinous cystic neoplasms of the pancreas. Jop. 2013;14(4):446-9. doi:10.6092/1590-8577/1402

51. Ballehaninna UK, Shaw JP, Brichkov I. Subdiaphragmatic bronchogenic cyst at the gastroesophageal junction presenting with Dysphagia: a case report. Surg Laparosc Endosc Percutan Tech. 2013;23(4):e170-2. doi:10.1097/SLE.0b013e31828e3e54

52. Kurokawa T, Yamamoto M, Ueda T, Enomoto T, Inoue K, Uchida A, et al. Gastric bronchogenic cyst histologically diagnosed after laparoscopic excision: report of a case. Int Surg. 2013;98(4):455-60. doi:10.9738/intsurg-d-12-00038.1

53. Castro R, Oliveira MI, Fernandes T, Madureira AJ. Retroperitoneal bronchogenic cyst: MRI findings. Case Rep Radiol. 2013;2013:853795. doi:10.1155/2013/853795

54. Runge T, Blank A, Schäfer SC, Candinas D, Gloor B, Angst E. A retroperitoneal bronchogenic cyst mimicking a pancreatic or adrenal mass. Case Rep Gastroenterol. 2013;7(3):428-32. doi:10.1159/000355879

55. Jannasch O, Büschel P, Wodner C, Seidensticker M, Kuhn R, Lippert H, et al. Retroperitoneoscopic and laparoscopic removal of periadrenally located bronchogenic cysts--a systematic review. Pol Przegl Chir. 2013;85(12):706-13. doi:10.2478/pjs-2013-0108

56. Terasaka T, Otsuka F, Ogura-Ochi K, Miyoshi T, Inagaki K, Kobayashi Y, et al. Retroperitoneal bronchogenic cyst: a rare incidentaloma discovered in a juvenile hypertensive patient. Hypertens Res. 2014;37(6):595-7. doi:10.1038/hr.2014.38

57. Maly T, Mihal V, Michalkova K, Tichy T, Neoral C, Zonca P. Retroperitoneal bronchogenic cyst: prenatal diagnosis of cystoid formation, its progression and surgery. Bratisl Lek Listy. 2014;115(2):98-100. doi:10.4149/bll_2014_021

58. Dong B, Zhou H, Zhang J, Wang Y, Fu Y. Diagnosis and treatment of retroperitoneal bronchogenic cysts: A case report. Oncol Lett. 2014;7(6):2157-9. doi:10.3892/ol.2014.1974

59. Cao DH, Zheng S, Lv X, Yin R, Liu LR, Yang L, et al. Multilocular bronchogenic cyst of the bilateral adrenal: report of a rare case and review of literature. Int J Clin Exp Pathol. 2014;7(6):3418-22.

60. Mirsadeghi A, Farrokhi F, Fazli-Shahri A, Gholipour B. Retroperitoneal bronchogenic cyst: a case report. Med J Islam Repub Iran. 2014;28:56.

61. Jiang X, Zeng H, Gong J, Huang R. Unusual uptake of radioiodine in a retroperitoneal bronchogenic cyst in a patient with thyroid carcinoma. Clin Nucl Med. 2015;40(5):435-6. doi:10.1097/rlu.0000000000000664

62. Altieri MS, Zheng R, Pryor AD, Heimann A, Ahn S, Telem DA. Esophageal bronchogenic cyst and review of the literature. Surg Endosc. 2015;29(10):3010-5. doi:10.1007/s00464-015-4082-4

63. Bulut G, Bulut MD, Bahadır I, Kotan Ç. Bronchogenic cyst mimicking an adrenal mass in the retroperitoneal region: report of a rare case. Indian J Pathol Microbiol. 2015;58(1):96-8. doi:10.4103/0377-4929.151200

64. Yoon YR, Choi J, Lee SM, Kim YJ, Cho HD, Lee JW, et al. Retroperitoneal Bronchogenic Cyst Presenting Paraadrenal Tumor Incidentally Detected by (18)F-FDG PET/CT. Nucl Med Mol Imaging. 2015;49(1):69-72. doi:10.1007/s13139-014-0306-0

65. Tong HX, Liu WS, Jiang Y, Liu JU, Zhou JJ, Zhang Y, et al. Giant retroperitoneal bronchogenic cyst mimicking a cystic teratoma: A case report. Oncol Lett. 2015;9(6):2701-5. doi:10.3892/ol.2015.3076

66. Leepalao M, Wernberg J. Multiple Bronchogenic and Gastroenteric Cysts Arising from the Stomach in a Patient with Abdominal Pain. Case Rep Surg. 2015;2015:601491. doi:10.1155/2015/601491

67. Trehan M, Singla S, Singh J, Garg N, Mahajan A. A Rare Case of Intra- Abdominal Bronchogenic Cyst- A Case Report. J Clin Diagn Res. 2015;9(11):Pd03-4. doi:10.7860/jcdr/2015/12949.6761

68. Zhang D, Zhang Y, Liu X, Zhu J, Feng C, Yang C, et al. Challenge in preoperative diagnosis of retroperitoneal mucinous cyst in a pediatric patient. Int J Clin Exp Med. 2015;8(10):19540-7.

69. Gou Y, Wang Y, Fang H, Xu X, Yu W, Zhang K, et al. Bronchogenic cyst in the hepatogastric ligament masquerading as an esophageal mesenchymal tumor: a case report. Int J Clin Exp Pathol. 2015;8(11):15307-11.

70. Herek D, Erbiş H, Kocyigit A, Yagci AB. Retroperitoneal Bronchogenic Cyst Originating from Diaphragmatic Crura. Indian J Surg. 2015;77(Suppl 3):1397-8. doi:10.1007/s12262-014-1045-2

71. Pasquer A, Djeudji F, Hervieu V, Rabeyrin M, Barth X. A rare retrorectal presentation of a bronchogenic cyst: A case report. Int J Surg Case Rep. 2016;24:112-4. doi:10.1016/j.ijscr.2016.05.028

72. Chhaidar A, Ammar H, Abdessayed N, Azzaza M, Gupta R, Abdennaceur N, et al. Large bronchogenic cyst of stomach: A case report. Int J Surg Case Rep. 2017;34:126-9. doi:10.1016/j.ijscr.2017.03.021

73. Gao X, Zhai M, Zhang H, Wang Y, Zhou J. A giant intradiaphragmatic bronchogenic cyst: case report and literature review. Tumori. 2017;103(Suppl. 1):e25-e7. doi:10.5301/tj.5000669

74. Wang M, He X, Qiu X, Tian C, Li J, Lv M. Retroperitoneal bronchogenic cyst resembling an adrenal tumor with high levels of serum carbohydrate antigen 19-9: A case report. Medicine (Baltimore). 2017;96(31):e7678. doi:10.1097/md.0000000000007678

75. Byers JT, Gertz HE, French SW, Wang L. Case report: Retroperitoneal bronchogenic cyst as a diagnostic dilemma after colon cancer diagnosis. Exp Mol Pathol. 2018;104(2):158-60. doi:10.1016/j.yexmp.2018.02.002

76. Liu Q, Gao Y, Zhao Z, Zhao G, Liu R, Lau WY. Robotic resection of benign nonadrenal retroperitoneal tumors: A consecutive case series. Int J Surg. 2018;55:188-92. doi:10.1016/j.ijsu.2018.04.013

77. Başoğlu M, Karabulut K, Özbalcı GS, Aykun N, Çamlıdağ İ, Güngör BB, et al. Laparoscopic resection of retroperitoneal bronchogenic cyst clinically presenting as adrenal cyst. Turk J Surg. 2018:1-3. doi:10.5152/turkjsurg.2018.4033

78. Chen HY, Fu LY, Wang ZJ. Ileal bronchogenic cyst: A case report and review of literature. World J Clin Cases. 2018;6(14):807-10. doi:10.12998/wjcc.v6.i14.807

79. Ye L, Yang D, Qin X, Hu B. An abdominal bronchogenic cyst. Gastroenterol Hepatol. 2020;43(3):140-1. doi:10.1016/j.gastrohep.2019.07.011

80. Venkatesh K, Pillarisetty K. Bronchogenic cyst presenting as content of omphalocele - A case report. Indian J Pathol Microbiol. 2020;63(1):116-8. doi:10.4103/ijpm.Ijpm_841_18

81. Wen Y, Chen W, Chen J, He X. Retroperitoneal bronchogenic cyst resembling an adrenal tumor: two case reports and literature review. J Int Med Res. 2020;48(5):300060520925673. doi:10.1177/0300060520925673

82. Bakshi N, Dhawan S, Sengar P, Dhir U. Neuroendocrine tumor in a lesser sac bronchogenic cyst - A hitherto unreported entity made rarer by concomitant hepatic hydatid cyst. Indian J Cancer. 2020;57(2):205-8. doi:10.4103/ijc.IJC_749_18

83. Subramanian JB, K SS, Selvarangam S. A case report- retroperitoneal bronchogenic cyst in relation to the hindgut. Int J Surg Case Rep. 2020;75:140-2. doi:10.1016/j.ijscr.2020.09.038

84. Qingyu J, Xiaolong L, Ruohan Z, Licong M, Zhichao T, Qingwei C, et al. Computed tomography helps pre-operative evaluation before laparoscopic resection of retroperitoneal bronchogenic cyst: A case report. J Minim Access Surg. 2021;17(1):95-7. doi:10.4103/jmas.JMAS_72_20

85. Sinha V, Nandi P, Shankar M, Sardana N. Retroperitoneal Bronchogenic Cyst: A Rare Case Study. Cureus. 2020;12(9):e10421. doi:10.7759/cureus.10421

86. Cowan S, Gunawardene A, Davenport E. Retroperitoneal bronchogenic cyst mistaken as an adrenal adenoma. ANZ J Surg. 2021;91(7-8):E526-e7. doi:10.1111/ans.16515

87. Cassiani J, Crinò SF, Manfrin E, Rivelli M, Gabbrielli A, Guglielmi A, et al. Endoscopic Ultrasound Through-the-Needle Biopsy for the Diagnosis of an Abdominal Bronchogenic Cyst. Clin Endosc. 2021;54(5):767-70. doi:10.5946/ce.2020.195

88. Hu L, Fan J, Wang T, Tang G. Ectopic Bronchogenic Cyst in Abdomen- A Rare Special Type. Am J Med Sci. 2021;361(6):e59-e60. doi:10.1016/j.amjms.2020.11.014

89. Tadokoro T, Misumi T, Itamoto T, Nakahara H, Matsugu Y, Ikeda S, et al. Retroperitoneal Bronchogenic Cyst Resected by Single-Incision Laparoscopic Surgery in an Adolescent Female: A Case Report. Asian J Endosc Surg. 2022;15(1):206-10. doi:10.1111/ases.12973

90. Wu LD, Wen K, Cheng ZR, Alwalid O, Han P. Retroperitoneal bronchogenic cyst in suprarenal region treated by laparoscopic resection: A case report. World J Clin Cases. 2021;9(24):7245-50. doi:10.12998/wjcc.v9.i24.7245

91. Yuan K, Shu M, Ma Y, Feng W, Ye J, Yuan Y. Ectopic bronchogenic cyst in the retroperitoneal region: a case report and literature review of adult patients. BMC Surg. 2021;21(1):347. doi:10.1186/s12893-021-01341-w

92. Kamimura G, Ueda K, Suzuki S, Aoki M, Nagata T, Sato M. A case of intradiaphragmatic bronchogenic cyst with an abnormally high serum level of CA19-9. Respirol Case Rep. 2021;9(10):e0838. doi:10.1002/rcr2.838

93. Clementino-Filho J, Surjan RCT, Taglieri E, Ardengh JC. Retroperitoneal Bronchogenic Cyst Mimicking a Pancreatic Cystic Lesion with Extremely High Level of Intralesional Fluid CA-19.9 Antigen: Benign in Disguise. Indian J Surg. 2021:1-6. doi:10.1007/s12262-021-03137-x

94. Hu BY, Yu H, Shen J. A retroperitoneal bronchogenic cyst clinically mimicking an adrenal mass: three case reports and a literature review. J Int Med Res. 2022;50(1):3000605211072664. doi:10.1177/03000605211072664

95. Toi N, Kurajoh M, Noda S, Emoto M. Retroperitoneal Bronchogenic Cyst Adjacent to Adrenocortical Adenoma. Intern Med. 2022. doi:10.2169/internalmedicine.9188-21
